# Gastric emptying is slow in chronic fatigue syndrome

**DOI:** 10.1186/1471-230X-4-32

**Published:** 2004-12-26

**Authors:** Richard B Burnet, Barry E Chatterton

**Affiliations:** 1 Department of Endocrinology and Metabolism, Royal Adelaide Hospital, North Terrace, Adelaide, South Australia 5000, Australia; 2 Department of Nuclear Medicine, Royal Adelaide Hospital, North Terrace, Adelaide, South Australia 5000, Australia

## Abstract

**Background:**

Gastrointestinal symptoms are common in patients with Chronic Fatigue Syndrome (CFS). The objective of this study was to determine the frequency of these symptoms and explore their relationship with objective (radionuclide) studies of upper GI function.

**Methods:**

Thirty-two (32) patients with CFS and 45 control subjects completed a questionnaire on upper GI symptoms, and the 32 patients underwent oesophageal clearance, and simultaneous liquid and solid gastric emptying studies using radionuclide techniques compared with historical controls.

**Results:**

The questionnaires showed a significant difference in gastric (p > 0.01) symptoms and swallowing difficulty. Nocturnal diarrhoea was a significant symptom not previously reported.

5/32 CFS subjects showed slightly delayed oesophageal clearance, but overall there was no significant difference from the control subjects, nor correlation of oesophageal clearance with symptoms. 23/32 patients showed a delay in liquid gastric emptying, and 12/32 a delay in solid gastric emptying with the delay significantly correlated with the mean symptom score (for each p ≪ 0.001).

**Conclusions:**

GI symptoms in patients with chronic fatigue syndrome are associated with objective changes of upper GI motility.

## Background

Chronic Fatigue Syndrome (CFS) is a descriptive term used to define a classifiable pattern of symptoms that cannot be attributed to any alternative condition [1]. It can be associated with immunological alterations, neuro-endocrine changes [2], sleep disturbance and disturbed neurocognitive performance with abnormal cerebral perfusion [3], but the pathophysiological significance of these is uncertain. Skeletal neuromuscular function is usually normal in CFS sufferers [4].

Many with CFS have gastro-intestinal (GI) symptoms, which are often unrecognised as being part of CFS. The commonest of the upper GI symptoms include fullness and bloating after a small meal, abdominal distension, nausea, and loss of appetite. Lower GI tract symptoms have considerable overlap with irritable bowel syndrome [5].

The hypothesis explored in this paper is that symptoms of possible upper gastrointestinal origin are more common in patients with CFS and are related to upper gastrointestinal motility as assessed by radionuclide methods.

## Methods

### Subjects

Consecutive patients with CFS who met the Fukuda criteria [6] for CFS were all seen by a single physician (RB). Patients with any medical condition which could account for chronic fatigue, a BMI > 30, previous GI surgery or medication affecting the rate of gastric emptying were excluded. Overt psychiatric disease was excluded at the interview. The patients were asked to self assess their percentage reduction in activity from prior to the onset of CFS as a marker of severity. Gastro-Intestinal symptoms were evaluated in patients and controls by a standard questionnaire prior to the gastric emptying studies [7].

Symptoms were divided into "**oesophageal**" (dysphagia, heart burn, acid regurgitation), "**gastric**": (anorexia, nausea, early satiety, bloating, abdominal distension, intermittent abdominal pain), "**other**" frequency of bowel actions, consistency of stools, presence or absence of diarrhoea, urgency and timing.

Symptoms were scored. 0, none, 1, mild (symptom could be ignored), 2, moderate (symptom could not be ignored, but did not influence daily activities), 3, severe, (symptom influenced daily activities). A mean symptom score (maximum score 3) for the 6 gastric symptoms, and 3 oesophageal symptoms was obtained.

The volunteer control subjects who completed the questionnaire were in regular full time employment, with no history of excessive fatigue, on no GI medication, and had no previous GI surgery.

### Radionuclide measurement of upper GI motility

Details and normal ranges of this double isotope test have been previously published [8]. The solid meal consisted of 100 g of cooked ground beef containing 40MBq in-vivo labelled ^99m^Tc-sulfur colloid-chicken liver, and the liquid meal consisted of 150 ml of 10% dextrose in water containing with 20 MBq of ^67^Ga-ethylenediaminetetraacetic acid (EDTA). All medication (except oral contraceptives) was discontinued for 24 hours prior to each study. The test was performed at 10 am (after an overnight fast) and monitored for at least two hours with the subject in the sitting position with the scintillation camera behind. The study commenced with a standardised oesophageal clearance study (solid bolus) followed by eating the solid meal and then immediately drinking the glucose solution. Each study was continued for at least 2 hours. Oesophageal clearance was expressed as time to 95% clearance (ref range < 93 sec) [9], Liquid gastric emptying as half-clearance time (ref 4–31 minutes) and solid emptying as amount remaining at 100 min (ref 4–61%).

The GI questionnaires were compared between CFS and control by Chi^2^, and Gastric emptying indices compared with historical normal range (t test comparison of means), and correlated with the mean symptom score (± SD).

The Study was approved by The Human Research Ethics Committee of the Royal Adelaide Hospital and informed consent given by the subjects.

## Results

Thirty-two (32) CFS patients (22F), with a mean age of 38.5 years had gastric emptying studies. Forty-five (45) control subjects undertook the questionnaire. The demographic details of the controls vs. patients are shown in table 1 Gastro-intestinal symptoms were more common in the CFS group (mean symptom score {MSS] 1.01 ± 0.87) than controls (MSS 0.24 ± 0.34) (table 2).

**Table 1:** Characteristics of CFS subjects vs controls. (SD)

	CFS	CONTROLS
Number	32	45
Sex	F 22, M10	F 37, M 8
Age (yr)	38.5 ± 13.9	34.4 ± 8.5
Weight Kg	68.1 ± 12.1	71.8 ± 11.6
Duration CFS (yr)	9.5 ± 6.8	
Severity, % reduction activity	65	
Smoke %	18	17.8

**Table 2:** Percentage frequency of any gastrointestinal symptoms.

	CFS % (n = 32)	CONTROLS % (n = 45)
**GASTRIC**		

Abdominal discomfort	39	22.
Fullness after small meal	70	31*
Nausea	67	15*
Abdominal pain	76	27*
Loss of appetite	42	12*
Vomiting	22	2*

**OESOPHAGEAL**		

Acid regurgitation	30	28
Heart burn	48	27
Swallowing difficulty	45	9*

**OTHER**		

Bowel movements/ day (mean)	1.6	1.2
Constipation %	26	30
Consistency Formed %	67	80
Loose/Watery %	33	20
Nocturnal diarrhoea %	21	0*
Faecal Urgency %	51	16*

* indicates symptoms more frequent in CFS group p <.05, Chi^2^

**Figure 1:**
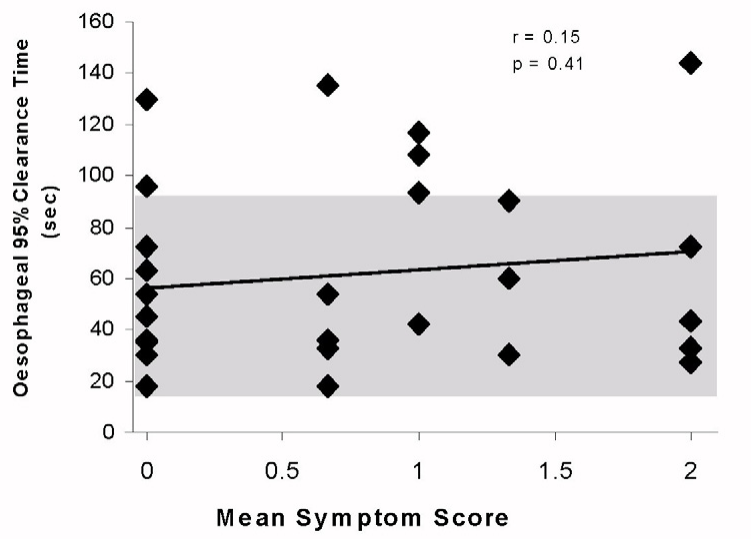
Oesophageal clearance time compared with oesophageal symptoms in Chronic Fatigue Syndrome. (Shaded area represents 95% confidence limit of normal reference range)

**Figure 2:**
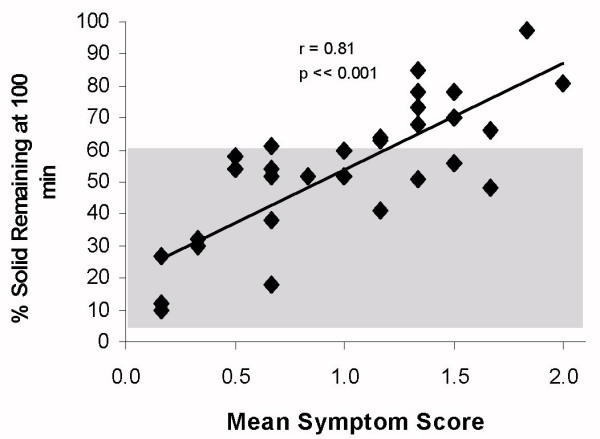
Per-cent gastric retention of solid food compared with mean symptom score in chronic fatigue syndrome. (Shaded area represents 95% confidence limit of normal reference range)

**Figure 3:**
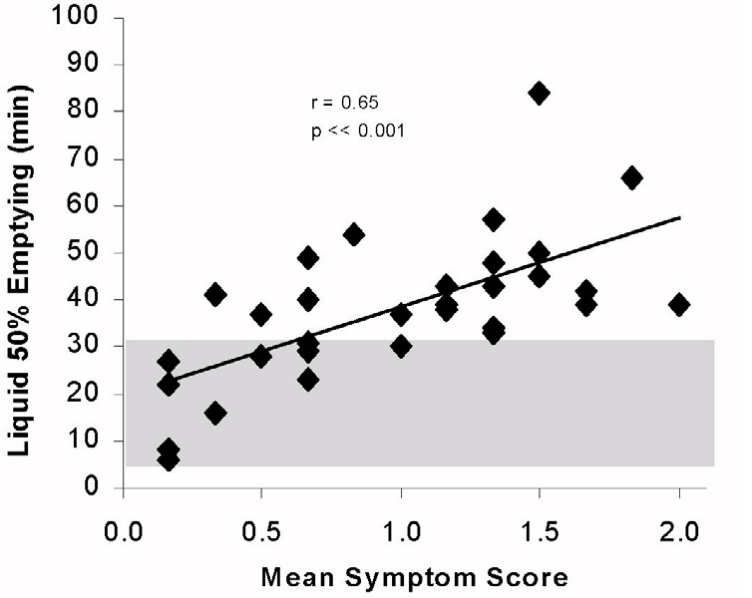
Time to 50% gastric emptying of liquid compared with mean symptom score in chronic fatigue syndrome. (Shaded area represents 95% confidence limit of normal reference range)

The overall, grouped gastric emptying studies of CFS subjects showed no significant slowing of oesophageal clearance p = 0.45 from the control population, and no significant correlation between emptying and oesophageal symptom score (r = 0.15) although 5 of the symptomatic and 2 of the asymptomatic subjects 7/32 (22%), were slower than the 95% confidence limits, (fig 1), this did not reach statistical significance. The major abnormality shown is a delay in the emptying of the liquid phase in 23/32 72% of the patients, whereas 12/32 (38%) of solid emptying was delayed compared with the historic controls (t comparison of means, figs 2 and 3, group p ≪ 0.005). When the gastric emptying results were compared to the mean symptom score there was a highly significant correlation of solid (r = 0.81) and liquid (r = 0.65) delay which increased with the symptom score (p ≪ 0.001).

## Discussion

G-I symptoms are common in patients with CFS. Abdominal pain is distressing [10], often requiring analgesia for relief. A previously unrecorded symptom in CFS patients is nocturnal diarrhoea, which disrupts an already disturbed sleep pattern. The most common upper GI symptom is fullness and bloating after a small meal. The usual medical explanation for the gut symptoms is 'irritable bowel'. Unless specific G-I questions are put to the CFS patient they will not spontaneously discuss these symptoms.

An abnormality in solid or liquid emptying or combinations of these study parameters was more common in the more symptomatic patients, and liquid was more frequently affected. This is the opposite of the abnormality seen in diabetic subjects [8], where the major abnormality, delay in the solid phase of gastric emptying has been ascribed to autonomic dysfunction or hyperglycaemia. A group of elderly subjects with a number of neurological defects showed a delay in the liquid rather than the solid emptying [11].

Symptoms and delayed gastric emptying in diabetic gastroparesis studies have not correlated well. In this study there is a good correlation with symptoms. The commonest of these was early satiety, fullness and bloating after eating. There was though a poor correlation with oesophageal symptoms and a disorder of oesophageal emptying.

GI motility is complex, with central, local neuromuscular and humoral influences. Non-specific endocrine disturbances have been demonstrated in CFS, but the relevance of these is unknown with regard to GI disturbances. Skeletal muscle fatigue appears to be of central rather than peripheral origin, but again it is not known whether this may be extrapolated to visceral muscle.

Inconclusive central changes have been documented. The impact of disturbed sensory function is unknown, and this could also involve peripheral nerves or the central processing of sensory information.

Diagnostically, there is overlap between CFS, functional dyspepsia and fibromyalgia and all may be related to abnormal sensory processing [10,12,13]. Altered gastric emptying has been shown in association with irritable bowel syndrome [14].

## Conclusions

These observations indicate that there is measurable disturbance in upper gut motility corresponding with symptoms in CFS. Although the cause for these findings is not apparent in this study, the more prominent delay in liquid rather than solid emptying may point to a central rather than a peripheral aetiology. The gastro-intestinal tract and function should be properly investigated and the symptoms not necessarily be ascribed to irritable bowel syndrome.

## Competing interests

The author(s) declare that they have no competing interests.

## Authors contribution

RB examined the patients and analysed the clinical data, BC performed the Nuclear Medicine studies, drafted the manuscript and performed the statistics. Both read and approved the final manuscript.

## Pre-publication history

The pre-publication history for this paper can be accessed here:


